# Role of frailty in otorhinolaryngology and head and neck surgery in a secondary and a tertiary care center: a prospective observational two-cohort study

**DOI:** 10.1038/s41598-025-34813-7

**Published:** 2026-01-06

**Authors:** Justus Herweg, Mohamed Nasreldin Mohamed, Katharina Geißler, Thomas Bitter, Eike Scholz, Orlando Guntinas-Lichius

**Affiliations:** 1 Department of Otorhinolaryngology, Jena University Hospital, Friedrich- Schiller-University Jena, Jena, Germany; 2 Department of Otorhinolaryngology, Head and Neck Surgery, Plastic Surgery, Voice and Speech Disorders, Gesundheitszentrum Bitterfeld/Wolfen, Bitterfeld-Wolfen, Germany

**Keywords:** Health care, Geriatrics, Risk factors

## Abstract

The proportion of elderly patients undergoing otolaryngology and head and neck surgery (OLHNS) is continuously increasing. The present prospective study evaluated whether frailty in patients ≥ 65 years of age was an important predictor of perioperative complications. An unselected series of 276 elderly patients (67% male; median age: 74 years; Charlson Comorbidity Index (CCI) 2+: 85%) undergoing OLHNS surgery in a 6-month period in 2022 and 2023, respectively, in a secondary and in a tertiary care hospital completed the Groningen Frailty Indicator questionnaire. Primary outcome measure was postoperative complications measured with the Clavien-Dindo classification (CDC), which were analyzed using univariate and multivariate binary logic regression models. 34.7% of the patients were frail. Female gender (Odds ratio [OR] = 2.158; confidence interval [CI] = 1.229 to 3.791; *p* = 0.007) and the CCI (OR = 1.258; CI = 1.135 to 1.396; *p* < 0.001) were independent predictors for frailty. 22.8% had CDC complications. Frailty (OR = 1.805; CI = 1.001 to 3.252; *p* = 0.049) and a CCI 2+ (OR = 4.821; CI = 1.102 to 21.079; *p* = 0.037) were independent predictors for postoperative CDC complications. The inclusion of a frailty assessment into the pre-operative risk assessment is recommended for all patients ≥ 65 years of age planned for OLHNS.

**Supplementary Information:**

The online version contains supplementary material available at 10.1038/s41598-025-34813-7.

## Introduction

The proportion of people aged 65 years and older is increasing at a faster rate than those below this age. The percentage of the global population aged 65 and above is expected to rise from 10% in 2022 to 16% in 2050^1^. In recent decades, the proportion of people aged 65 and older has been increasing more steadily in rural areas than in urban areas, and the more sparsely populated the districts are, the greater the increase of the proportion of elderly people^2^. One consequence of this trend is the increasing number of hospitalized older and very old otorhinolaryngology patients^3^. This demographic shift could have a significant impact on the preoperative risk assessment for older patients undergoing otorhinolaryngology and head and neck surgeries. Traditionally, the assessment relies on patient age, comorbidities, surgical extent, and the patient’s performance status. These factors are used to predict whether a patient is at risk of adverse perioperative outcomes such as increased length of stay, complications, and mortality^4^. Prediction of perioperative risk based on physiological reserve or frailty is still not part of routine clinical practice. Frailty is defined as a syndrome of age-related decline in physical, cognitive, social, and psychological functioning, along with a reduced ability of the body to cope with increased stress. As a result, frailty leads to a diminished physiological reserve across multiple organ systems^5,6^. Several frailty classification systems have been published. The Groningen Frailty Indicator (GFI), a self-assessment questionnaire, can easily be used in primary care and hospital care settings^7^. The GFI has already been utilized in head and neck cancer patients^4,8,9^. So far, a frailty assessment has been less common used in pre-operative risk assessments for non-cancer otolaryngology and head and neck surgeries^10^.

Hence, the aim of the present prospective study was to explore the role of frailty in an unselected series of otolaryngology and head and neck surgeries. Furthermore, the impact of an increasing number of older otolaryngology patients in rural settings was assessed by including patients from a secondary care hospital with a rural catchment area, and comparing the results to patients from a tertiary care hospital with a state-wide catchment area. The primary outcome parameter was the occurrence of postoperative complications.

## Materials and methods

### Study design and setting

This prospective observational two-cohort study was conducted in two departments of Otorhinolaryngology, Head and Neck Surgery. The department in Bitterfeld is a secondary care center located in a small town with a rural catchment area. The department in the town Jena is a tertiary and university care center with a state-wide catchment area. The study was conducted in both centers over 6 months. It ran from January 2022 to July 2022 in the tertiary care hospital, and then from May 2023 to November 2023 in the secondary care hospital.

### Inclusion criteria

All patients admitted to the departments were screened. During the 6-month period, 131 patients aged ≥ 65 years were admitted for surgery to the secondary care hospital and 196 patients to the tertiary care hospital. Hence, 327 patients fulfilled the inclusion criteria. There were no exclusion criteria. All these patients were asked participate in the study and to complete the frailty questionnaire. Of these, 276 patients (inclusion rate of 84%) agreed to participate. Four patients declined to participate in the secondary care hospital and 47 patients in the tertiary care center. The patients’ characteristics of the participants were compared to those of the non-participants (Supplemental Table 1). Non-participants were more prevalent in the tertiary hospital (*p* < 0.001). Non-participating patients were younger (*p* = 0.022) and had a lower comorbidity (*p* = 0.010). Among the group of participants were more patients with a benign tumors, more infectious diseases, and sensory/functional disabilities (*p* = 0.037). Other differences were not observed.

### Assessment of the frailty using the Groningen frailty indicator

The 15-item Groningen Frailty Indicator (GFI) questionnaire is used to screen for self-reported limitations. The GFI includes four frailty domains of frailty: physical components (mobility, comorbidity, physical energy, vision, and hearing), psychological component (depressed mood and anxiety feelings), cognitive component (cognition) and social component (loneliness). A score of 1 or 0 is assigned to any ‘yes/sometimes’ or ‘no’ response, respectively. Higher scores indicate higher levels of frailty and an increased need for integrated care^7^. A total score of ≤ 3 represents a non-frail patient. A total score of ≥ 4 indicates frailty^11^. The validated German version of the GFI was used^12^.

### Other clinical variables

Demographic and medical variables were measured by chart review. The Charlson comorbidity index (CCI) was used to measure the patients’ general comorbidity^13^. Management of complication was recorded and graded according to the surgical Clavien-Dindo Classification (CDC)^14^. The CDC score classifies complications into five groups. CDC grading is based on the severity of the intervention required for the complication. In cases involving multiple complications, the CDC grading is based on the most extensive intervention.

### Statistics

All statistical analyses were performed using IBM SPSS Statistics 25 (Chicago, IL). Nominal and ordinal data are presented as absolute values.Relative values areexpressed as percentages. The results of the metric parameters are presented as means ± standard deviation (SD), median and range, unless otherwise indicated. The Chi square test and nonparametric Mann–Whitney U test were used to compare the characteristics of two independent subgroups (non-frail versus frail patients or patients with versus without postoperative complication). Spearman’s correlation analysis was used to analyze the correlation of metric/ordinal parameters. Multivariate binary logistic regression models with stepwise entry were generated for the analysis of independent factors associated with an increased risk of frailty and CDC complications (CDC ≥ I). Patients’ characteristics used in the regression analyses were derived from the variables that were significant in the preliminary univariate analyses (*p* < 0.05). In general, nominal p values of two-tailed tests are reported. P values < 0.05 were considered significant.

### Ethical approval

Approval for the study was obtained through the local institutional ethics review board, the Ethics committee of the Friedrich-Schiller-University, Jena, Germany (Nr. 2022-2509-Bef). Written informed consent was obtained from all study participants. All experimental procedures involving human participants adhered to the ethical standards of the institutional research committee and the 1964 Helsinki Declaration and its subsequent amendments.

## Results

### Comparison of the participating frail patients versus non-frail patients

34.7% of the patients were categorized as frail. All comparisons are shown in Table 1. Frail patients (median: 77 years, range: 65–95) were older than the non-frail patients (median: 72 years, range: 65–92 years; *p* = 0.002). Age showed a moderate direct linear correlation with the frailty index (*r* = 0.230, *p* < 0.001). Frail patients were more frequently women (*p* = 0.025) and had a higher comorbidity score (*p* < 0.001). The Charlson Comorbidity Index showed a direct linear correlation to the Groningen Frailty Index (*r* = 0.352; *p* < 0.001). Hospital stay was longer in frail compared to non-frail patients (*p* = 0.010). Table 2 gives an overview on the postoperative complications measured with the CDC in frail and non-frail patients. More CDC complications were observed in frail patients (*p* = 0.016). This difference was mainly due to a higher number of CDC I and CDC II complications in frail patients than in non-frail patients. However, when using CDC III as the cut-off point, this difference between frail and non-frail patients disappeared. Hence, the frequency of higher CDC scores did not differ between both groups. Multivariate analysis revealed that female gender (odds ratio [OR] = 2.158; confidence interval [CI] = 1.229 to 3.791; *p* = 0.007) and the comorbidity index (OR = 1.258; CI = 1.135 to 1.396; *p* < 0.001) were independent predictors of frailty (Table 3).

**Table 1:** Patients‘ characteristics of frail and non-frail patients.*.

Parameter	Frail		Non-frail		*p*
	*N*	%	*N*	%	
All	96	100	180	100	
Hospital setting					0.834
Secondary	45	46.9	82	45.6	
Tertiary	51	53.1	98	54.4	
Gender					**0.025**
Male	56	58.2	129	71.7	
Female	40	41.7	51	28.3	
Surgery site					0.086
Nose	5	5.2	13	7.2	
Oral cavity	15	15.6	8	4.4	
Ear/Vestibular	11	11.5	31	17.2	
Neck	13	13.5	18	10.0	
Eye	4	4.2	5	2.8	
Face	0	0.0	2	1.1	
Nasopharynx	0	0.0	2	1.1	
Larynx/Trachea	8	8.3	26	14.4	
Paranasal sinus	5	5.2	5	2.8	
Oropharynx	2	2.1	10	5.6	
Salivary glands	9	9.4	19	10.6	
Skin	18	18.8	34	18.9	
Hypopharynx	2	2.1	4	2.2	
Esophagus	3	3.1	2	1.1	
Thyroid	0	0.0	1	0.6	
Other	1	1.0	0	0.0	
Surgery type					0.692
Minor surgery	44	45.8	87	48.3	
Major surgery	52	54.2	93	51.7	
Malignant tumor					0.069
No	45	46.9	105	58.3	
Yes	51	53.1	75	41.7	
Disease classification					0.267
Malignant tumor	51	53.1	75	41.7	
Benign tumor/mass	14	14.6	41	22.8	
Trauma/Bleeding	1	1.0	5	2.8	
Infection/Inflammation	13	13.5	32	17.8	
Sensory/functional impairment	17	17.7	26	14.4	
Other	0		1	0.6	

	M ± SD	Median; range	M ± SD	Median; range	
Age in years	75.9 ± 8.8	77; 65–95	74.0 ± 6.7	72; 65–92	**0.002**
Charlson comorbidity index	5.6 ± 3.3	5; 0–13	3.7 ± 2.6	3; 0–13	**< 0.001**
Length of stay in days	6.3 ± 7.9	4; 2–66	4.6 ± 2.9	4; 1–27	**0.010**

Groningen Frailty Indicator ≥ 4 = frail patients; M = mean; SD = standard deviation.

**Table 2:** Postoperative CDC complication in frail and non-frail patients.*.

Parameter	Frail		Non-frail		*p*
	*N*	%	*N*	%	
All	96	100	180	100	
CDC classification					**0.016**
No complication	65	67.7	148	82.2	
CDC I	15	15.6	20	11.1	
CDC II	9	9.4	4	2.2	
CDC IIIa	1	1.0	0	0	
CDC IIIb	3	3.1	8	4.4	
CDC IVa	1	1.0	0	0	
CDC IVb	1	1.0	0	0	
CDC V	1	1.0	0	0	
CDC complication categorized I					**0.006**
No complication (CDC 0)	65	67.7	148	82.2	
Complication (CDC I+)	31	32.3	32	17.8	
CDC classification categorized II					**0.009**
CDC 0/I	80	83.3	168	93.3	
CDC II+	16	16.7	12	6.7	
CDC classification categorized III					0.320
CDC 0/I/II	89	92.7	172	95.6	
CDC III+	7	7.3	8	4.	

Groningen Frailty Indicator ≥ 4 = frail patients; M = mean; SD = standard deviation; CDC = Clavien-Dindo classification.

**Table 3:** Multivariate analysis of the associations between patients’ characteristics and higher probability to be a frail patient than a non-frail patient.

Measure	OR	95% CI lower	95% CI upper	*p*
Gender				
Male	1	Reference		
Female	2.158	1.229	3.791	**0.007**
Age in years	1.009	0.971	1.048	0.647
Charlson comorbidity index	1.258	1.135	1.396	**< 0.001**

Multivariable binary logistic regression for the dichotomized outcome parameter non-frail patients versus frail patient. Significant p-values (*p* < 0.05) in bold. OR = odds ratio; CI = confidence interval.

### Other factors with association to higher probability of postoperative complications

**Fig. 1:**
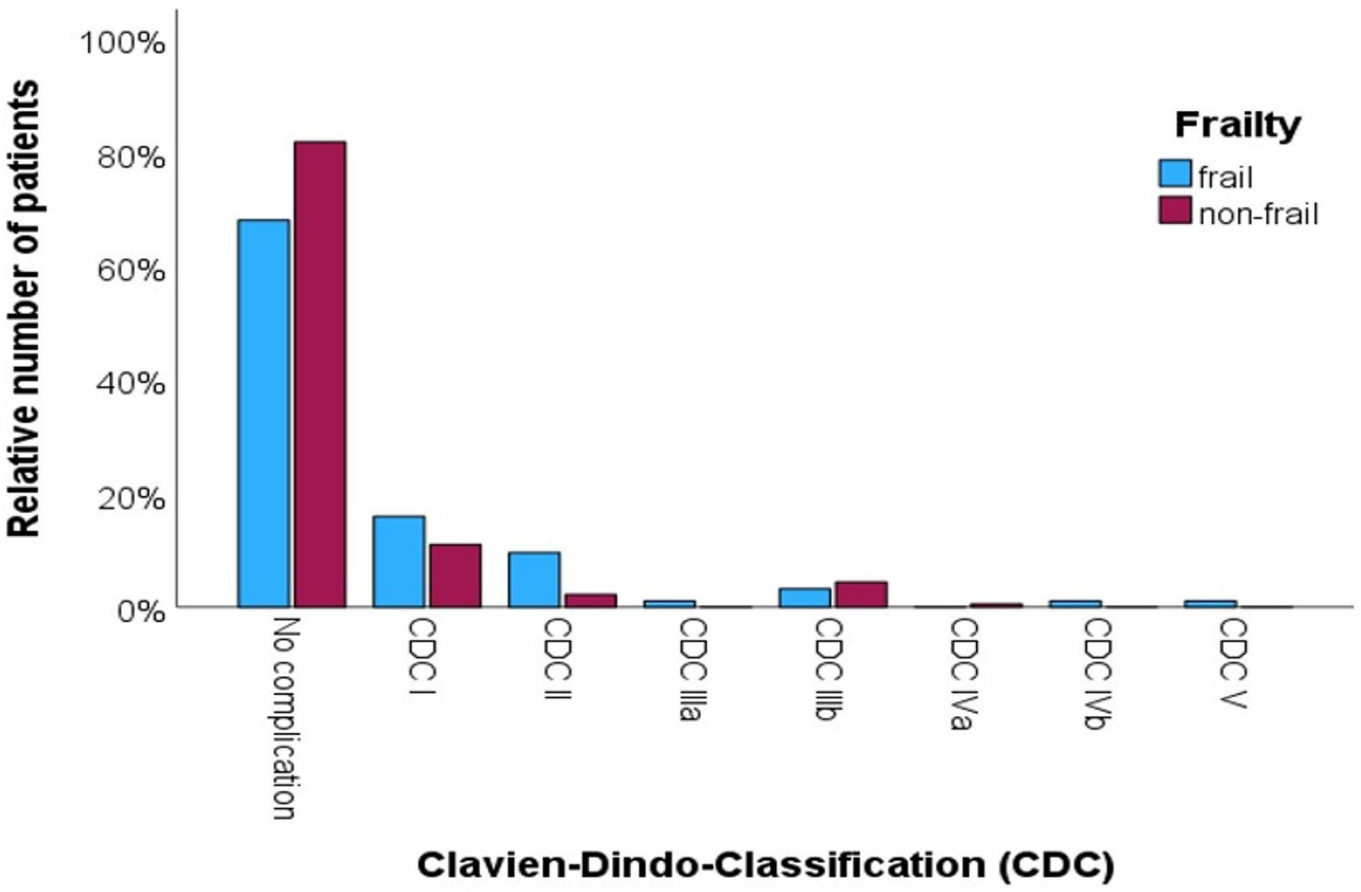
Postoperative Clavien-Dindo classification (CDC) complications in frail (blue) and non-frail (red) patients.

22.8% of patients experienced CDC complications. Table 4 shows details of the comparison between patients with and without postoperative CDC complications. Figure 1 illustrates the distribution of CDC complications in frail and non-frail patients in. CDC complications were observed in 49.2% and 30.5% of frail and non-frail patients, respectively. A higher number of complications (CDC I+) were observed in patients undergoing surgery for non-malignant diseases (*p* = 0.008), with higher comorbidity (CCI 2+; *p* = 0.003), and frailty (*p* = 0.006). Multivariate analysis revealed that frailty (OR = 1.805; CI = 1.001 to 3.252; *p* = 0.049) and CCI 2 + comorbidity (OR = 4.821; CI = 1.102 to 21.079; *p* = 0.037) as independent predictors for postoperative CDC complications (Table 5). A higher CDC classification correlated with a longer hospital stay (*r* = 0.302; *p* < 0.001).

**Table 4:** Comparison of patients without and with postoperative CDC complications.

Parameter	No complication (CDC 0)		Complications (CDC I+)		*p*
	*N*	%	*N*	%	
All	213	100	63	100	
Hospital setting					0.563
Secondary	96	45.1	31	49.2	
Tertiary	117	54.9	32	50.8	
Gender					0.814
Male	142	66.7	43	68.3	
Female	71	33.3	20	31.7	
Surgery site					0.492
Nose	16	7.5	2	3.2	
Oral cavity	14	6.6	9	14.3	
Ear/Vestibular	37	17.4	5	7.9	
Neck	26	12.2	5	7.9	
Eye	7	3.3	2	3.2	
Face	2	0.9	0	0.0	
Nasopharynx	2	0.9	0	0.0	
Larynx/Trachea	22	10.3	12	19.0	
Paranasal sinus	8	3.8	2	3.2	
Oropharynx	8	3.8	4	6.3	
Salivary glands	21	9.9	7	11.1	
Skin	39	18.3	13	20.6	
Hypopharynx	5	2.3	1	1.6	
Esophagus	4	1.9	1	1.6	
Thyroid	1	0.5	0	0.0	
Other	1	0.5	0	0.0	
Malignant tumor					**0.008**
Yes	125	58.7	25	39.7	
No	88	41.3	38	60.3	
Disease classification					0.115
Malignant tumor	88	41.3	38	60.3	
Benign tumor/mass	43	20.2	12	19.0	
Trauma/Bleeding	5	2.3	1	1.6	
Infection/Inflammation	40	18.8	5	7.9	
Sensory/functional impairment	36	16.9	7	11.1	
Other	1	0.5	0	0	
Charlson Comorbity Index (CCI)					**0.003**
CCI 0/1	39	18.3	2	3.2	
CCI 2+	174	81.7	61	96.8	
Frailty					**0.006**
Yes	65	30.5	31	49.2	
No	148	69.5	32	50.8	

	M ± SD	Median; range	M ± SD	Median; range	
Age in years	75.1 ± 7.54	74; 65–93	74.7 ± 8.2	72; 65–95	0.761
Charlson comorbidity index	4.1 ± 2.8	4; 0–13	5.4 ± 3.4	5; 0–13	**0.002**
Length of stay in days	3.0 ± 2.6	2; 0–12	4.0 ± 3.0	3; 0–11	**0.015**

M = mean; SD = standard deviation.

**Table 5:** Multivariate analysis of the associations between patients’ characteristics and higher probability of postoperative CDC complications (CDC I+) compared to no complications.

Measure	OR	95% CI lower	95% CI upper	*p*
Malignant tumor				
No	1	Reference		
Yes	1.783	0.989	3.216	0.055
Frailty				
No	1	Reference		
Yes	1.805	1.001	3.252	**0.049**
Charlson comorbidity index				
CCI 0/1	1	Reference		
CCI 2+	4.821	1.102	21.079	**0.037**

Multivariable binary logistic regression for the dichotomized outcome parameter CDC (no CDC complication (CDC 0) versus CDC complication (CDC I+). Significant p-values (*p* < 0.05) in bold. OR = odds ratio; CI = confidence interval; CCI = Charlson Comorbidity Index.

### Comparison of the participating patients treated in a secondary care setting versus a tertiary setting

All data on the comparison can be found in Supplement Table 2. More salivary gland diseases were identified in the secondary care setting, whereas oropharyngeal and neck diseases were more frequently identified in the tertiary setting (*p* = 0.003). Sensory/functional disorders were more prevalent in the tertiary setting (*p* = 0.009). Age (*p* < 0.001) and comorbidity (*p* < 0.001) were higher in the secondary care setting with rural catchment area. There was no difference in the frailty rate between the two settings (*p* = 0.834). Postoperative complications, as measured with the CDC classes II and IIIb, were more frequent in the tertiary center (*p* = 0.018), though the overall CDC complication rate did not differ significantly(*p* = 0.563).

## Discussion

Head and neck surgeons and anesthesiologists have predominantly used chronological age and comorbidity to assess perioperative risk in head and neck cancer and other otolaryngology patients^15^. Frailty among elderly head and neck cancer patients is now recognized as a distinct clinical entity that is widely prevalent^16^. This was confirmed in the present study not only for patients with head and neck cancer (45.6% of the study population), but also, for the first time, for an unselected series covering all common types of otolaryngology and head and neck cancer surgeries. Gender and comorbidity but not age were independent predictors for frailty. Moreover, frailty was also distinct from the biological age in terms of the primary outcome of the present study. Frailty and comorbidity, but not age, were independent predictors of postoperative complications.

Frailty screening tools typically detect between 11 and 69% of frail patients in cohorts of patients aged ≥ 65 years with primary head and neck cancer^16,17^. In the present study, 34.7% of the patients were frail, i.e., the frailty rate was in the lower range of that observed in other studies. The chosen screening tool and the types of diseases included may have influenced the frailty rate. We assume that the frailty rate is higher overall in study cohorts that include only patients with malignant tumors, compared to unselected series such as the present study, which also included patients undergoing other otorhinolaryngology procedures. In the present study, there was a non-significant trend toward higher frailty prevalence in patients with malignant tumors than in other patients. We actually hypothesized that the patient cohort treated in the secondary care hospital in a rural area would have a higher proportion of frail patients than the cohort from the tertiary hospital in an urban setting^18^. This could not be confirmed. As expected, the patients from the rural area were older and had more comorbidities, but frailty did not differ. In general, a rural area is defined as a geographic area located outside towns and cities, although there is no international consensus on this definition. Typical rural areas have low population densities and small settlements. The secondary care hospital analyzed in the present study is located in a small town of around 37,000 inhabitants. The surrounding area is rural, but patients from the town itself are also treated there. The differences in frailty might have been greater if the two patient cohorts had been dichotomized according to exact place of residence rather than treating hospital. We are not aware of any comparable Western studies focusing on surgical scenarios, but the data from mainly Asian studies show an ambivalent picture regarding frailty in the general population: some studies report of higher frailty in rural communities, while others do not (see examples^18–20^:). Whether the setting (rural or urban community) plays a role in frailty and surgical complications requires further investigation.

In general, frail patients are more likely to experience complications^21^. In the case of head and neck oncologic surgery, frailty is associated with an increased risk of CDC IV and V complications (with a CDC IV and V complication rate of 26–47% in frail patients)^4,17^. The present study included all types of otorhinolaryngology, head and neck cancer surgery, covering a wide range of procedures from minor to major surgery. Overall, 22.8% of the patients in the present study experienced CDC complications. The complication rate (CDC ≥ grade I) was 17.8% in the non-frail patients and 32.3% in the frail patients. Hence, the complication rate was approximately doubled in frail patients undergoing all types of otorhinolaryngology and head and neck surgery. However, it should be noted that these were predominantly minor complications (CDC grades I and II). Severe complications (CDC grade III+) were rare overall. The CDC complication rate after unselected head and neck surgery can vary from 14% to high rates to as high as 64% in high-risk surgery involving free flaps after major head and neck surgery^22^. In the present trial, comorbidity and frailty but not age were independent risk factors for complications after surgery. This underlines once again underlines that frailty is an independent construct separate from age and comorbidity.

Two methodological questions must be discussed: Firstly, there is no consensus on which frailty measure to use in a preoperative setting. Over 70 frailty assessments have been described in the literature, but many of these have not been extensively validated^15^. The validated GFI is easy to implement in daily practice. Many see the Comprehensive Geriatric Assessment (CGA) as the gold standard in identifying frailty in elderly patients, particularly cancer patients^23^. However, the CGA has to be carried out by a medical specialist and a geriatric team of allied health professionals. This makes it personnel-intensive and time-consuming, which in turn hampers its widespread implementation as preoperative risk assessment tool on a large scale. We can determine that the GFI was easy to use in both the secondary and tertiary hospital setting. Whether the CGA would lead to different results could only be determined by a much more elaborate study that used both the CGA and the GFI to classify patients. Secondly, the conventional cut-off value of 4 for the GFI was adopted from previous studies^7,24,25^. However, a different cut-off value may be more sensitive in predicting the risk of surgical complications for patients undergoing OLHNS^26^. This must be investigated in future studies.

The present study had limitations. Firstly, frailty is a multidimensional concept involving many physical, psychological and social aspects of health. As previously mentioned, the CGA is the most comprehensively researched model for healthcare delivery to frail older patients and is primarily employed on geriatric wards^27^. As emphasized several times, the GFI used in the present study is a screening tool. Hence, unlike the CGA, it does not reflect all dimensions of frailty in detail. Secondly, although there were only a few differences in characteristics between participants and non-participants, a selection bias cannot be completely ruled out. The patients were younger in the non-participating group, mainly because the rate of young-old patients (aged 65–70 years) was higher in this group. Furthermore, the participation was lower in the tertiary setting. Finally, there are several other potential perioperative parameters associated with postoperative complications were not analyzed in the present study (such as surgical duration, type/extent of surgery, and the ASA classes). These factors must be considered as potential sources of bias when interpreting the data. For instance, longer surgery time is a general risk factor for postoperative complications, independent of the surgical discipline^28–30^.

Head and neck surgery, especially head and neck cancer surgery, are complex. Multidisciplinary management is clinical routine^31^. The GFI requires only minimal training and takes less than 2–3 min to complete. A viable option to integrate the GFI into preoperative pathways could be to administer the tool to the nursing staff during the admission process or to the anesthetic staff alongside ASA grading, with automatic flagging of frail patients for multidisciplinary team review together with the geriatricians^32,33^.

## Conclusions

The present study has shown that assessing frailty is valuable not only for head and neck cancer surgery but also for otorhinolaryngology and head and neck surgery in general. Frailty was found to be associated with a higher risk of postoperative complications. Therefore, a frailty assessment should be integrated into the routine preoperative assessment of patients aged over 65 years. However, the question remains as to which prophylactic preoperative measures can be taken for frail patients scheduled for otorhinolaryngology and head and neck surgery in order to reduce the risk of postoperative complications.

## Supplementary Information

Below is the link to the electronic supplementary material.

Supplementary Material 1

## Data Availability

The original contributions presented in the study are included in the article and in the supplementary material. Further inquiries can be directed to the corresponding author.
