# Supplementary material for: Role of frailty in otorhinolaryngology and head and neck surgery in a secondary and a tertiary care center: a prospective observational two-cohort study
Source: Sci Rep. 2026 Jan 6;16:635. doi: 10.1038/s41598-025-34813-7 (PMC12775424; doi:10.1038/s41598-025-34813-7)
Supplement: Supplementary file 1 — Supplementary Material 1 [file 41598_2025_34813_MOESM1_ESM.docx]

**Role of frailty in otorhinolaryngology and head and neck surgery in a secondary and a tertiary care center: a prospective observational two-cohort study**

Justus Herweg, Mohamed Nasreldin Mohamed, Katharina Geißler, Thomas Bitter, Eike Scholz, Orlando Guntinas-Lichius

**Supplemental Tables**

**Supplemental Table 1**

| **Supplemental Table 1.** Comparison of the participants and the non-participants. | | | |
| --- | --- | --- | --- |
| **Parameter** | **Participants** | **Non-participants** | **p** |
| All | 276 | 51 |  |
| Hospital setting |  |  | **<0.001** |
| Secondary care | 127 | 4 |  |
| Tertiary care | 149 | 47 |  |
| Gender |  |  | 0.552 |
| Male | 185 | 32 |  |
| Female | 91 | 19 |  |
| Surgery site |  |  | 0.195 |
| Nose | 18 | 0 |  |
| Oral cavity | 23 | 2 |  |
| Ear/Vestibular | 42 | 9 |  |
| Neck | 31 | 7 |  |
| Eye | 9 | 1 |  |
| Face | 2 | 0 |  |
| Lung | 0 | 1 |  |
| Nasopharynx | 2 | 0 |  |
| Larynx/Trachea | 34 | 13 |  |
| Paranasal sinus | 10 | 4 |  |
| Oropharynx | 12 | 1 |  |
| Salivary glands | 28 | 4 |  |
| Skin | 52 | 8 |  |
| Hypopharynx | 6 | 1 |  |
| Esophagus | 5 | 0 |  |
| Thyroid | 1 | 0 |  |
| Other | 1 | 0 |  |
| Malignant tumor |  |  | 0.942 |
| Yes | 150 | 28 |  |
| No | 126 | 23 |  |
| Disease classification |  |  | **0.037** |
| Malignant tumor | 126 | 23 |  |
| Benign tumor/mass | 55 | 5 |  |
| Trauma/Bleeding | 6 | 0 |  |
| Infection/Inflammation | 45 | 6 |  |
| Sensory/functional impairment | 43 | 16 |  |
| Other | 1 | 1 |  |
|  | **M±SD** | **M±SD** | **p** |
| Age in years | 75.0±7.7 | 72.3±6.4 | **0.022** |
| Charlson Comorbidity Index (CCI) | 4.4±3.0 | 3.2±2.7 | **0.010** |
| Length of stay in days | 5.2±5.2 | 6.4±5.8 | 0.125 |

M= mean; SD = standard deviation

**Supplemental Table 2**

| **Supplemental Table 2.** Comparison of the participants in the secondary and the tertiary care hospital. | | | |
| --- | --- | --- | --- |
| **Parameter** | **Secondary care hospital** | **Tertiary care hospital** | **p** |
| All | 127 | 149 |  |
| Gender |  |  | 0.823 |
| Male | 86 | 99 |  |
| Female | 41 | 50 |  |
| Surgery site |  |  | **0.003** |
| Nose | 9 | 9 |  |
| Oral cavity | 10 | 13 |  |
| Ear/Vestibular | 25 | 17 |  |
| Neck | 10 | 21 |  |
| Eye | 2 | 7 |  |
| Face | 1 | 1 |  |
| Lung | 0 | 2 |  |
| Nasopharynx | 15 | 19 |  |
| Larynx/Trachea | 6 | 4 |  |
| Paranasal sinus | 5 | 7 |  |
| Oropharynx | 7 | 21 |  |
| Salivary glands | 36 | 16 |  |
| Skin | 1 | 5 |  |
| Hypopharynx | 0 | 5 |  |
| Esophagus | 0 | 1 |  |
| Thyroid | 0 | 1 |  |
| Other | 9 | 9 |  |
| Malignant tumor |  |  | 0.089 |
| No | 62 | 88 |  |
| Yes | 65 | 61 |  |
| Disease classification |  |  | **0.009** |
| Malignant tumor | 65 | 61 |  |
| Benign tumor/mass | 26 | 29 |  |
| Trauma/Bleeding | 2 | 4 |  |
| Infection/Inflammation | 25 | 20 |  |
| Sensory/functional impairment | 9 | 34 |  |
| Other | 0 | 1 |  |
| Clavien-Dindo classification (CDC) |  |  | **0.018** |
| No complication | 96 | 117 |  |
| CDC I | 22 | 13 |  |
| CDC II | 1 | 12 |  |
| CDC IIIa | 1 | 0 |  |
| CDC IIIb | 4 | 7 |  |
| CDC IVa | 1 | 0 |  |
| CDC IVb | 1 | 0 |  |
| CDC V | 1 | 0 |  |
| CDC complication categorized |  |  | 0.563 |
| No complication (CDC 0) | 96 | 117 |  |
| Complication (CDC I+) | 31 | 32 |  |
| Frailty |  |  | 0.834 |
| Frail | 45 | 51 |  |
| Non-frail | 82 | 98 |  |
|  | **M±SD** | **M±SD** | **p** |
| Age in years | 76.9±8.3 | 73.4±6.5 | **<0.001** |
| Charlson Comorbidity Index (CCI) | 6.3±2.8 | 2.7±2.1 | **<0.001** |
| Groningen Frailty Indicator (GFI) | 3.2±2.9 | 3.2±2.9 | 0.962 |
| Length of stay in days | 5.5±7.0 | 4.9±3.0 | 0.378 |

M= mean; SD = standard deviation
